# Evaluating ensemble learning approaches for horizontal gene transfer detection

**DOI:** 10.1038/s41598-026-53037-x

**Published:** 2026-05-28

**Authors:** Andre Jatmiko Wijaya, Aleksandar Anžel, Georges Hattab

**Affiliations:** 1 Center for Artificial Intelligence in Public Health Research (ZKI-PH), Robert Koch Institute, 13353 Berlin, Germany; 2 Department of Mathematics and Computer Science, Freie Universität Berlin, 14195 Berlin, Germany

**Keywords:** Computational biology and bioinformatics, Genetics

## Abstract

Horizontal gene transfer (HGT) is widely recognized as a major driver of antimicrobial resistance (AMR) dissemination, with genomic islands (GIs) as one of the drivers facilitating the spread. Detecting GIs is essential for improving AMR surveillance. Numerous computational approaches have been developed for GIs detection, including recent advances in machine learning (ML). Several studies in other fields have shown that ML model performance depends on data representations. Combining multiple data representations in ensemble learning has been shown to improve performance in other genomics tasks. However, this approach has not yet been evaluated for GIs detection. To this end, we investigate the efficacy of integrating diverse data representations in ensemble learning for GIs detection, particularly for classification task. Then, we assess its applicability to localizing GIs, which are clusters of genes acquired through HGT, in a genomic sequence. We implemented a two-stage ensemble selection strategy to determine the optimal combination of data representations. Our ensemble selection strategy reveals that combining low-correlated data representations in an ensemble classifier yields a slightly higher Recall than individual representation for the classification task, but the improvement is not statistically significant. Nevertheless, the ensemble classifier could not localize GIs better, suggesting that the cross-task generalizability remains constrained. This finding presents an opportunity for future research to advance the field by redefining the problem formulation of GIs detection.

## Introduction

Horizontal gene transfer (HGT) is a pivotal evolutionary process in bacteria through which genetic material is transferred between organisms via mechanisms other than parent-offspring inheritance. HGT plays a critical role in shaping microbial genomes, driving adaptation, and facilitating the spread of traits, such as antimicrobial resistance (AMR)^1–3^. A significant part of the HGT is or has been facilitated by genomic islands (GIs)^4^. Mobile genetic elements (MGEs), such as plasmids, prophages, and integrative elements, are known to facilitate the mobilization of genomic islands (GIs), distinct chromosomal regions acquired through HGT. These GIs typically encode novel functions, including virulence factors and AMR genes^5,6^. Detecting GIs is therefore essential for understanding genome evolution, microbial ecology, and public health. Furthermore, tracking the transmission factors that contribute to the dissemination of AMR genes improves AMR surveillance systems^7^.

Existing approaches are primarily based on compositional analysis, comparative genomics, or a combination of the two, with recent advances in machine learning approaches^8^. We recently conducted a comprehensive study on data representations for ML in HGT detection^9^. Our study demonstrated that the efficacy of machine learning (ML) in detecting horizontal gene transfer (HGT) is influenced by data representations. One notable data representation was identified for HGT detection. However, we also identified other data representations. These exhibited low correlation with the predominant data representation. Yet they demonstrated comparable efficacy. This suggests that integrating these data representations could enhance the learning of HGT patterns. Depending on the functions in a genome, genomic information is encoded on different scales, from ten to mega base pairs^10^. This genomic information can be quantified by different data representations, for instance, GC content. By viewing genomic sequence through multiple representational lenses, we can capture a more integrated and holistic picture of its structure and signals. Combining multiple data representations enables models to leverage complementary information, mitigating the risk of overlooking critical signals and enhancing prediction accuracy in tasks such as enhancer prediction, methylation site identification, and genomic variant classification^11–13^. Furthermore, integrating various data representations from different properties, including composition- and physicochemical-based properties, has been shown to improve the prediction of antimicrobial peptides (AMP)^14^. This finding suggests that a similar strategy could be effective in detecting HGT.

There are two approaches to combining data representations: hybrid and fusion^15^. Hybrid approaches combine multiple data representations into a single feature set to train a single model. Fusion approaches use separate models for each data representation and then combine their outputs using ensemble learning. A prior study combined different data representations as features to train an SVM model, demonstrating that a hybrid approach is essential for detecting a wider range of HGT events^16^. Ensemble learning integrates the strengths of several models to produce more reliable and accurate predictions, offering a potentially effective solution. Studies have shown that ensemble models outperform individual models in various biological tasks^17–19^. However, no study has evaluated the effectiveness of ensemble learning for HGT detection.

In this study, we investigated the effectiveness of using an ensemble learning approach to combine multiple data representations, particularly low-correlated and high-performing ones, for GIs detection. Our study focused on detecting GIs as a distinct chromosomal region within a genome, rather than specific MGEs. We used 44 data representations and five ML models as base classifiers for the ensemble classifier. Due to the large number of data representations and base classifiers utilized, we employed a two-stage ensemble selection strategy. First, we pruned the candidates for the ensemble classifiers. A “candidate” is a base classifier trained on a dataset encoded by a specific data representation. Then, we applied an ensemble growing method to select the optimal set of candidates. We compared different ensemble methods and examined the difference between using heterogeneous and homogeneous base classifiers. Finally, we evaluated the ensemble classifier’s performance on classification and GI boundaries prediction tasks. Our study demonstrated the value of low-correlated data representations using ensemble learning. We identified the need to reframe the problem formulation for HGT detection. The current approach, which divides the problem into classification and boundary prediction tasks, has demonstrated limitations. This is because models trained for classification are not well-suited for whole-genome scanning^20^.

## Results

We present our results in the upcoming sections by following these steps. First, we trained five base classifiers for each data representation. Then, we performed cross-validation to determine the best classifier for each representation. Next, we combined the data representations into ensemble classifiers using a two-stage ensemble selection strategy. The first stage prunes candidates for an ensemble classifier. The second stage searches for the optimal subset of the remaining candidates. Third, we evaluated the best ensemble classifier and compared it with the best base classifier. Finally, we assessed the performance of the best ensemble classifier on the GI boundaries prediction task. We conducted our study using the Benbow dataset.

### Performance of different data representations and classifiers

To understand the performance of different data representations and classifiers, we cross-validated diverse combinations of data representations and classifiers (see Methods for more details). We performed a 5-fold StratifiedGroupKFold cross-validation, which we repeated 5 times, and reported the average Matthew correlation coefficient (MCC) for each combination of data representation and classifier. Stratification was done at the species level to ensure distinct sets of species in the training and validation datasets.

**Fig. 1:**
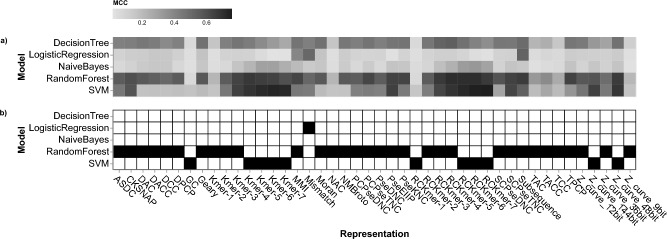
(**a**) Cross-validation results for various model and representation combinations. The color coding represents the Matthew correlation coefficient (MCC). The darker the color, the higher the value. (**b**) The selected model for each data representation is shown. Black boxes represent the selected classifier for each data representation. The x-axis displays the data representations and the y-axis depicts the base classifiers.

Figure 1a shows a heatmap of the cross-validation results, which corresponds to Fig. 1a from our previous study^9^. We found that random forest (RF) is more robust to data representation than other classifiers. However, SVM outperforms RF in certain data representations. For k-mer-based representations, RF performance declines from k-mer 4 to k-mer 7, while SVM performance increases as the k-mer size increases. This suggests that SVM is more robust for longer data representations than RF. To avoid using the same data representation, we selected the best-performing classifier for each representation, as shown in Fig. 1b. Ultimately, there are 44 candidates, which is equivalent to the number of data representations.

### Ensemble classifiers

A two-stage ensemble selection strategy was implemented to construct ensemble classifiers. First, the pool of candidates for the ensemble classifier was reduced to narrow the search. Second, an ensemble growth strategy was used to select the optimal subset of candidates.

The pruning process was formulated as a multi-objective optimization problem with two objectives: maximizing diversity and minimizing the error rate. Diversity was measured using Cohen’s kappa agreement^21^, while the error rate was quantified by $1-MCC$. Two pruning solutions, Pareto frontier (pfront) and convex hull (chull), were applied by following a study on encoding selection with ensemble pruning^15^.

**Fig. 2:**
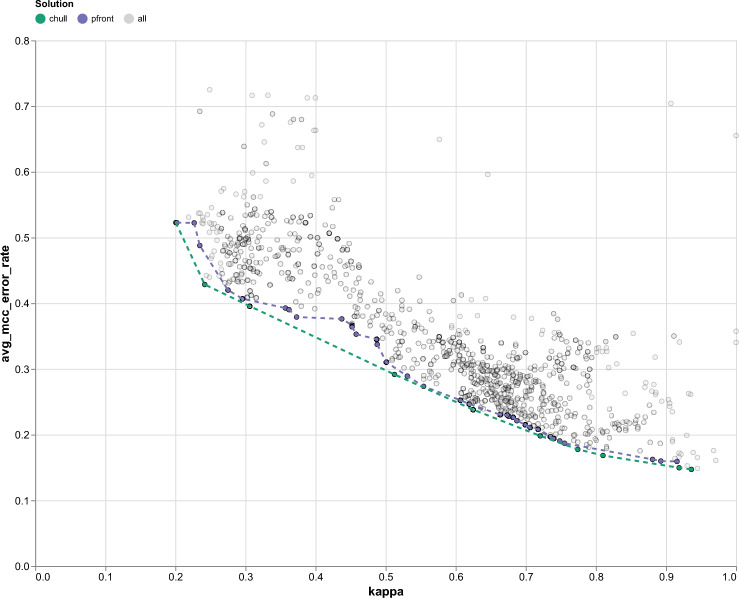
Example of a Kappa-error diagram from one fold in cross-validation. Each dot represents a pair of candidates (one data representation and one classifier). The gray dots correspond to all possible pairs of candidates. The green dots represent pairs selected by the convex hull, and the purple dots represent pairs selected by the Pareto frontier. The optimal solution for the ensemble classifier should have a low average error rate and low Kappa, indicating high diversity, and is located in the lower left part of the diagram. The error rate is defined as $1-MCC$, where MCC is Matthew correlation coefficient. An average error rate is the average error rate of each candidate in a pair.

Figure 2 shows an example of a kappa-error diagram, where the x-axis represents the kappa agreement between pairs of candidates and the y-axis shows the average error rate between them. We provided the kappa-error diagrams of all cross-validation folds in Supplementary Fig. S1 for visual comparison only.

Since there were potentially many candidates, we limited the number to 20. In addition to the pfront and chull solutions, Spänig et al.^15^ selected the 15 best-performing encodings. We selected the top 20 candidates as the optimal subset solely based on their performance (MCC) in the cross-validation results; thus, we used 3 solutions to select the candidates: pfront, chull, and best. After narrowing down the candidates, we used an ensemble growing procedure to select the optimal subset for the ensemble classifier. We applied 3 ensemble methods: *voting_soft*, *voting_hard*, and *stacking*.

**Fig. 3:**
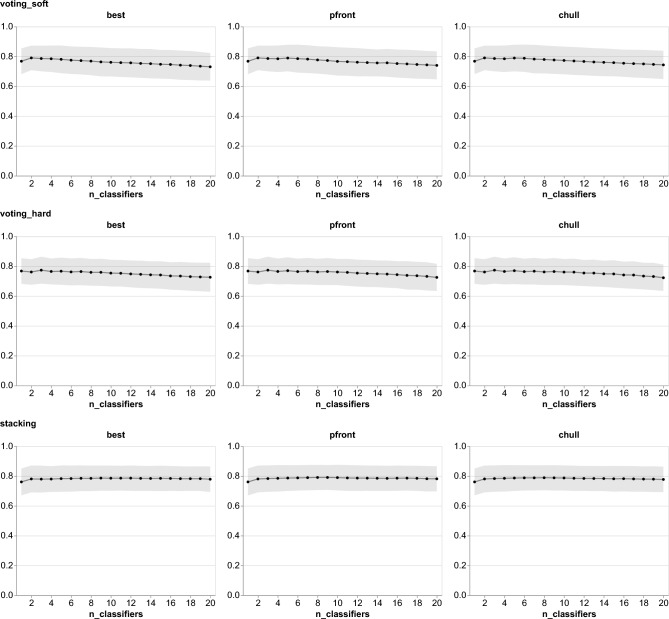
Ensemble learning performance is evaluated with different numbers of base classifiers across different ensemble methods and solutions. *voting_soft*, *voting_hard*, and *stacking* ensemble methods were used. For the ensemble solutions, we used Pareto frontiers (pfront), convex hulls (chull), and the best base classifiers to select candidates for ensemble learning. The solid line shows the mean Matthew correlation coefficient (MCC), and the shaded band represents the standard deviation across cross-validation folds. Five-fold cross-validation was repeated five times.

Figure 3 illustrates the cross-validation results for ensemble classifiers that have different numbers of base classifiers. On the one hand, we observed a similar trend in ensemble classifiers with voting strategies, wherein the performance increased when the number of base classifiers was increased up to a certain value (2 and 3 for *voting_soft* and *voting_hard*, respectively), and subsequently decreased as the number of base classifiers grew. On the other hand, the performance of ensemble classifiers with *stacking* exhibited relative stability irrespective of the size of the ensemble classifier. This phenomenon can be attributed to the use of a meta learner in the *stacking* method. A meta learner can combine the predictions of multiple base classifiers. Ensemble classifiers with voting, on the other hand, depend on the performance of base classifiers.

**Table 1:** Selected classifiers by different ensemble solutions and methods. All ensemble classifiers are better than the best base classifier, but corrected repeated k-fold cv test^22^ indicated no statistical difference between them, except the *voting_soft* method. After applying the Bonferroni-Holm correction^23^, which provided adjusted p-values, we noticed there is no statistical difference between all of them.

Method	Solution	Candidates	MCC	*p*-value	adj. *p*-value
*stacking*	best	RCKmer-7/SVM, Subsequence/RF, RCKmer-6/SVM, PseEIIP/SVM, RCKmer-4/RF, CKSNAP/SVM, Kmer-3/SVM, Z_curve_48bit/SVM, Kmer-7/SVM	0.788	0.475	1.0
	chull	RCKmer-7/SVM, Subsequence/RF, GC/SVM, RCKmer-6/SVM, Kmer-1/RF, RCKmer-4/RF	0.788	0.346	1.0
	pfront	RCKmer-7/SVM, Subsequence/RF, GC/SVM, RCKmer-6/SVM, Kmer-1/RF, RCKmer-4/RF, PseEIIP/SVM, Z_curve_48bit/SVM	0.789	0.346	1.0
*voting_hard*	best	RCKmer-7/SVM, RCKmer-6/SVM, Subsequence/RF	0.773	0.286	1.0
	chull	RCKmer-7/SVM, RCKmer-6/SVM, Subsequence/RF	0.773	0.286	1.0
	pfront	RCKmer-7/SVM, RCKmer-6/SVM, Subsequence/RF	0.773	0.286	1.0
*voting_soft*	best	RCKmer-7/SVM, Subsequence/RF	0.789	0.033	0.297
	chull	RCKmer-7/SVM, Subsequence/RF	0.789	0.033	0.297
	pfront	RCKmer-7/SVM,Subsequence/RF	0.789	0.033	0.297

Table 1 shows the selected candidates for each ensemble method and solution that yields the best ensemble model. We observed that RCKmer-7 with SVM and subsequence with random forest were selected in all methods and solutions. RCKmer-7 with SVM was selected because it performed the best individually, whereas subsequence with random forest did not perform as well as RCKmer-7 with SVM. Among other candidates with similar performance, subsequence with random forest was selected because it offered higher diversity. Thus, when both candidates were combined in an ensemble classifier, they performed better than they did individually.

In comparison to voting strategies, the *stacking* method achieved the best performance with relatively more classifiers. Although adding more classifiers in ensemble learning with *stacking* gives an improvement, the gain is not as much as adding one classifier to the best base classifier; thus, similar performance can be achieved with fewer classifiers. Furthermore, we conducted a statistical test, namely the corrected repeated k-fold cv test^22^, to compare the performance of the best base classifier and the ensemble classifiers. The statistical test indicated that there is no significant difference between the base best classifier and all ensemble classifiers, except the *voting_soft* ensemble ($p=0.033$). After applying the Bonferroni-Holm correction, we found no ensemble classifier statistically outperforming the single classifier.

**Fig. 4:**
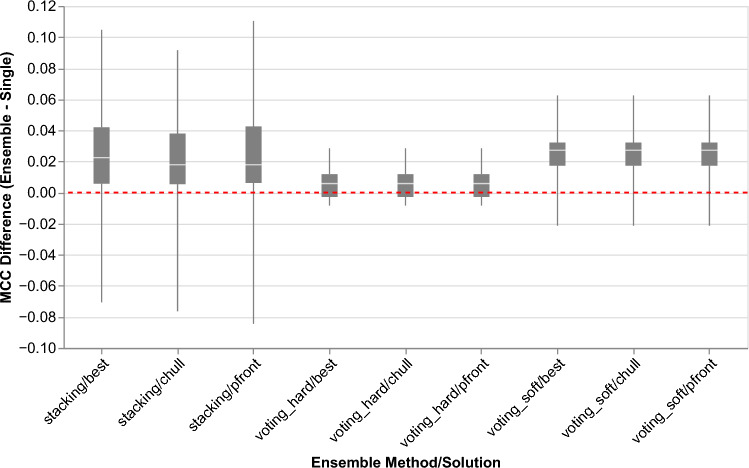
Difference performance between single and ensemble classifiers across cross-validation folds for all ensemble methods and solutions. Differences were calculated on the same fold.

Additionally, we analyzed the performance differences measured by MCC between single and ensemble classifiers across all cross-validation folds, as depicted in Fig. 4. The *stacking* ensemble demonstrated the highest variance in performance differences, while the *voting_hard* ensemble showed the least variance. The *voting_soft* ensemble exhibited intermediate variance but achieved the highest average performance differences compared to the single classifier. Consequently, the *voting_soft* ensemble (comprising RCKmer-7/SVM and Subsequence/RF) was chosen as the ensemble classifier for further analysis in this study, where it was compared to the best single classifier (RCKmer-7/SVM).

### Heterogeneous vs homogeneous base classifiers

Our approach to ensemble selection differs from Spänig et al.^15^, which combines different data representations with the same base classifier. Here, we investigated whether combining data representations with heterogeneous base classifiers is better than with homogeneous base classifiers. We performed the two-stage ensemble selection strategy on 5 base classifiers and compared the results with our study.

**Fig. 5:**
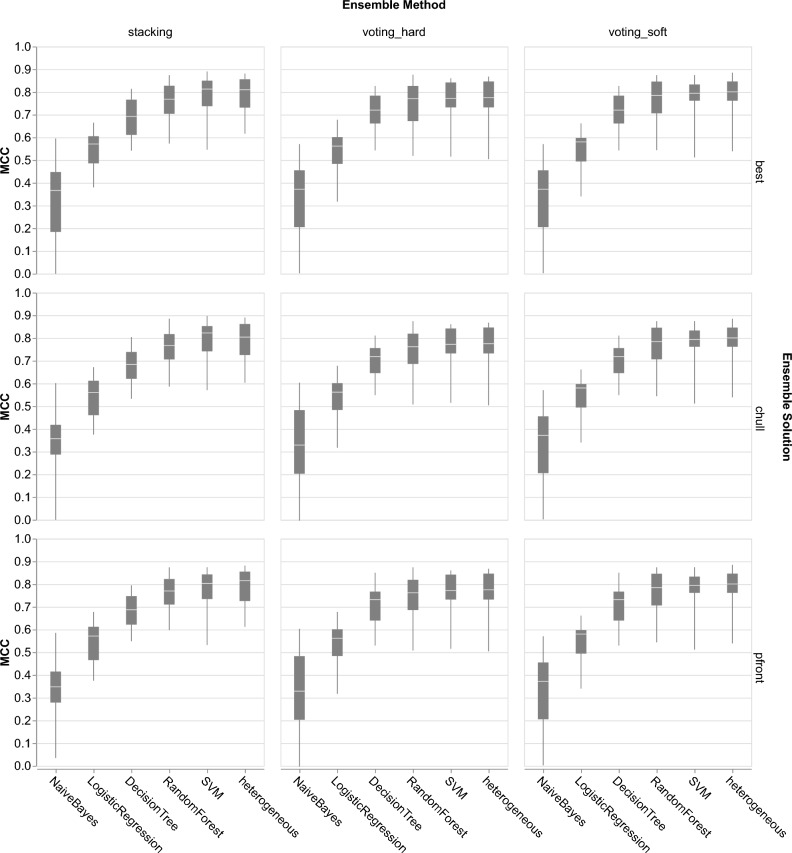
A comparison of Matthew correlation coefficient (MCC) between ensemble classifiers with two different base classifiers types: heterogeneous and homogeneous. Each row represents the ensemble solutions (pareto frontier, convex hull, and best), and each column displays the ensemble methods (*stacking*, *voting_hard*, *voting_soft*). Heterogeneous base classifiers significantly outperformed homogeneous base classifiers across all ensemble methods and solutions (corrected repeated k-fold cv test^22^ $p<0.05$), except decision tree, random forest, and SVM.

The boxplot in Fig. 5 shows a comparison of ensemble and homogeneous base classifiers. Across all ensemble methods and solutions, heterogeneous base classifiers demonstrated higher performance levels than homogeneous base classifiers. However, corrected repeated k-fold cv tests^22^ with Bonferroni-Holm correction indicated that heterogeneous base classifiers significantly outperformed homogeneous base classifiers across all ensemble methods and solutions, except Decision Tree, Random Forest, and SVM ($p>0.05$).

### Evaluate ensemble classifier

After finding the best ensemble classifier, we compared it with the best single classifier on two tasks, which are classification and GI boundaries prediction task. We trained both classifiers on the entire Benbow training dataset and evaluated them using the Benbow test dataset, which consists of 20 genomes. Then, we used the same trained classifiers on the GI boundaries prediction task. Additionally, we reported the performance of another candidate in the ensemble classifier, which is Subsequence/Random Forest.

Initially, we selected the *voting_soft* ensemble method, but it performed sub-optimally in the boundary prediction task compared to the single classifier, despite outperforming it in the classification task. As an alternative, we also evaluated the *stacking* ensemble method. We compared the performance of the single classifier, ensemble classifier with *voting_soft*, and *stacking* on the entire Benbow test dataset.

#### Evaluation on classification task

**Fig. 6:**
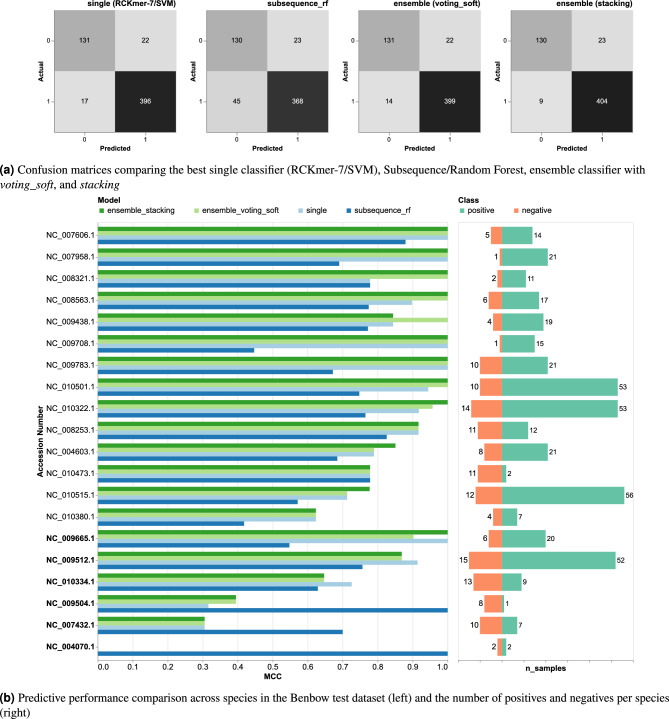
Comparison of the best single classifier (RCKmer-7/SVM), Subsequence/Random Forest, ensemble classifier with *voting_soft*, and *stacking* on the Benbow test dataset. (**a**) Confusion matrices showed both ensemble classifiers captured more positives than the single classifier. 0 is the negative class and 1 is the positive class. (**b**) The left figure shows the Matthew correlation coefficient (MCC) of each classifier across 20 species, whereas the right figure shows the number of positive and negative samples per species. The ensemble classifiers are better than the single classifier on some species, but they were outperformed by single classifiers on 6 species (NC_009665.1, NC_009512.1, NC_010334.1, NC_009504.1, NC_007432.1, and NC_004070.1).

Figure 6a shows confusion matrices of all classifiers. Both ensemble classifiers were more effective in capturing positive instances compared to the single classifier, with the *stacking* ensemble method identifying even more positives than the *voting_soft* method. Furthermore, we analyzed their performance across 20 species in the test dataset. The left bar chart in Fig. 6b shows the MCC comparison of all classifiers across 20 species, whereas the right bar chart displays the number of positives and negatives per species. The ensemble classifiers were overall superior to the single classifiers, but they were outperformed by either the single classifier or Subsequence/Random Forest on 6 species (NC_009665.1, NC_009512.1, NC_010334.1, NC_009504.1, NC_007432.1, and NC_004070.1). NC_009665.1 and NC_010334.1 are representatives of the genus *Shewanella*, identified as *Shewanella baltica* OS185 and *Shewanella halifaxensis* HAW-EB4; NC_009512.1 corresponds to *Pseudomonas putida* F1; NC_009504.1 is *Shewanella halifaxensis* ATCC 25840 chromosome II; NC_007432.1 and NC_004070.1 belong to genus *Streptococcus* (*Streptococcus agalactiae* A909 and *Streptococcus pyogenes* MGAS315, respectively). Replacing *voting_soft* with *stacking* showed both improvement and a drop in performance on certain species. None of the ensemble classifiers could successfully distinguish positive from negative classes for NC_004070.1 (*Streptococcus pyogenes* MGAS315), which had the fewest samples.

Overall, the ensemble classifiers (Recall: 0.966 and 0.978 for *voting_soft* and *stacking*, respectively) achieved higher recall than the single classifier (Recall: 0.959), highlighting its ability to capture positive classes better. However, it is noteworthy that there is a different positive class ratio between training and test datasets. The test set contains 566 sequences from 20 genomes, with 413 positives (73.0% positive) compared to 1,329 positives out of 2,569 sequences (51.7% positive) in the training of 147 genomes. This 21.3% point shift toward higher positive prevalence may influence Recall estimates, as models trained on more balanced data can appear to have higher Recall when evaluated on a positive-enriched dataset. To address this, we provided bootstrapped confidence intervals on all evaluation metrics in Table 2, where the ensemble consistently outperforms the single classifier in Recall (0.979 vs 0.959), F1 score (0.962 vs 0.953), MCC (0.854 vs 0.824), and Accuracy (0.944 vs 0.931), except Precision (0.946 vs 0.947), all with 95% bootstrap confidence intervals. These increases in prevalence-robust metrics suggest that the benefits of the ensemble method surpass the effects of distribution shifts that favor the Recall. However, McNemar’s exact test on per-sample paired predictions (n=566 samples) indicated no significant difference between single and ensemble classifiers ($p=0.18$ and $p=0.07$ for *voting_soft* and *stacking*, respectively). Notably, the ensemble classifier identified 9 samples that were missed by the single classifier, while there were 6 and 2 samples (*voting_soft* and *stacking*, respectively) with errors that were unique to the ensemble. This improvement might seem negligible, but this is highly important for the GIs detection.

**Table 2:** Bootstrapped performance metrics (95% confidence intervals) comparing single classifier vs ensemble on held-out test set (n=566 samples, 73.0% positive prevalence).

Metric	Single	Ensemble (*voting_soft*)	Ensemble (*stacking*)
MCC	0.824 [0.764, 0.875]	0.837 [0.785, 0.887]	0.854 [0.803, 0.901]
F1 score	0.953 [0.937, 0.967]	0.957 [0.942, 0.969]	0.962 [0.946, 0.975]
Precision	0.947 [0.923, 0.966]	0.948 [0.926, 0.968]	0.946 [0.923, 0.967]
Recall	0.959 [0.939, 0.977]	0.966 [0.947, 0.983]	0.979 [0.964, 0.991]
Accuracy	0.931 [0.910, 0.950]	0.936 [0.913, 0.956]	0.944 [0.924, 0.961]

Bootstrapped 95% CIs (B=1000 resamples) from held-out test set, keeping trained models fixed

*MCC* Matthews correlation coefficient

#### Evaluation on boundaries prediction task

To test the performance of the ensemble classifier to localize GIs, we adopted an existing pipeline for predicting GI boundaries developed by Banerjee et al.^24^ and replaced the ML model with the ensemble and single classifiers. The existing pipeline uses a sliding window approach to scan a genome and predict GI boundaries. The pipeline determines the confidence of its classification of a window as a GI segment depending on the probability of that window. To assign a class to each window, it applies a lower and upper threshold. The lower threshold is set to 0.5 and the upper threshold is set to 0.8. If the probability of a given window falls below the lower threshold, the window is labeled as the negative class and it is not processed further. Otherwise, if the probability exceeds this upper threshold, the pipeline can classify it as the positive class. Any windows with probabilities between the lower and upper thresholds will undergo further processing of a boundary refinement step (see Fig. 3 in^24^). To ensure a fair comparison, we used the default hyperparameters of the pipeline with a window size of 10,000 base pairs (bp), a lower threshold of 0.5, and an upper threshold of 0.8.

In addition to the comparison between the best ensemble and single classifiers, we provided a comparison with other baselines that were reported in our previous study^9^. Predictions of other baselines are available in https://github.com/FriedbergLab/GenomicIslandPrediction/tree/master/evaluation/predictions^24^. We additionally trained the single classifier (RCKmer-7/SVM) with the best hyperparameters found in our previous study (kernel: ’rbf’, C: 2, gamma: ’scale’). To make a fair comparison, we also trained both ensemble classifiers (*voting_soft* and *stacking*) with the best hyperparameter for RCKmer-7/SVM, but used default hyperparameters for Subsequence/RF. Table 3 shows the evaluation results on 20 genomes in the Benbow test dataset. We observed that the ensemble classifier *voting_soft* (default hyperparameters) performed the worst in the boundaries prediction task in all evaluation metrics, with MCC of 0.22, F1 score of 0.31, Precision of 0.99, Recall of 0.21, and Accuracy of 0.51. This finding contradicts the evaluation results on the classification task, demonstrating that the ensemble classifier is slightly better than the single classifier. However, the ensemble classifier *stacking* (default hyperparameters) was slightly better than the single classifier with MCC of 0.59, F1 score of 0.72, Precision of 0.92, Recall of 0.71, and Accuracy of 0.81. It achieved higher Recall (0.71 vs 0.67) and lower Precision (0.92 vs 0.95) than the single classifier (default hyperparameters). Overall, the single classifier with the best hyperparameters surpassed all other baselines in nearly all evaluation metrics, except Precision. Furthermore, integrating the single classifier with the best hyperparameters and subsequence/RF using default hyperparameters into an ensemble did not enhance performance.

**Table 3:** Evaluation metrics achieved by the single and ensemble classifiers (with *voting_soft* and *stacking*) as well as other baselines for GIs detection are listed by descending Matthew correlation coefficient (MCC). Each metric is averaged across 20 species in the Benbow Test dataset. All evaluation results of other baselines are available in https://github.com/FriedbergLab/GenomicIslandPrediction/tree/master/evaluation/predictions^24^.

Predictor	MCC	F1 score	Precision	Recall	Accuracy
Single (fine-tuned)*	**0.68**	**0.81**	0.88	**0.84**	**0.84**
Ensemble (*stacking* fine-tuned)*	0.66	0.80	0.88	0.83	0.83
IslandViewer4	0.60	0.81	0.95	0.75	0.81
TreasureIsland	0.59	0.78	0.93	0.71	0.82
Ensemble (*stacking*)	0.59	0.72	0.92	0.71	0.81
Single	0.56	0.70	0.95	0.67	0.82
IslandPick	0.33	0.45	**1.00**	0.37	0.63
Islandpath DIMOB	0.29	0.48	0.93	0.37	0.57
Ensemble (*voting_soft* fine-tuned)*	0.29	0.39	0.99	0.28	0.54
AlienHunter	0.28	0.62	0.80	0.55	0.62
SIGI-HMM	0.28	0.38	0.98	0.26	0.51
Islander	0.21	0.28	**1.00**	0.19	0.48
Ensemble (*voting_soft*)	0.22	0.31	0.99	0.21	0.51

*Fine-tuned hyperparameters were only for RCKmer-7/SVM (kernel:’rbf’, C: 2, gamma:’scale’), obtained by our previous study^9^, but default hyperparameters for Subsequence/RF (n_estimators:100, criterion:’gini’, max_samples_split:2, min_samples_leaf:1, max_features: ’sqrt’).

Significant values are in bold.

To understand the discrepancy between the evaluation results on the classification and the boundaries prediction tasks, we examined the performance of the single and ensemble classifiers under different conditions. We varied the window size from 5,000 to 15,000 with a 1,000 step and the threshold from 0.6 to 0.8 with a 0.1 step. Then, we compared the single classifier with the ensemble classifiers (*voting_soft* and *stacking*) using default hyperparameters. Additionally, we made a similar comparison of classifiers with fine-tuned hyperparameters (see Supplementary Fig. S2).

**Fig. 7:**
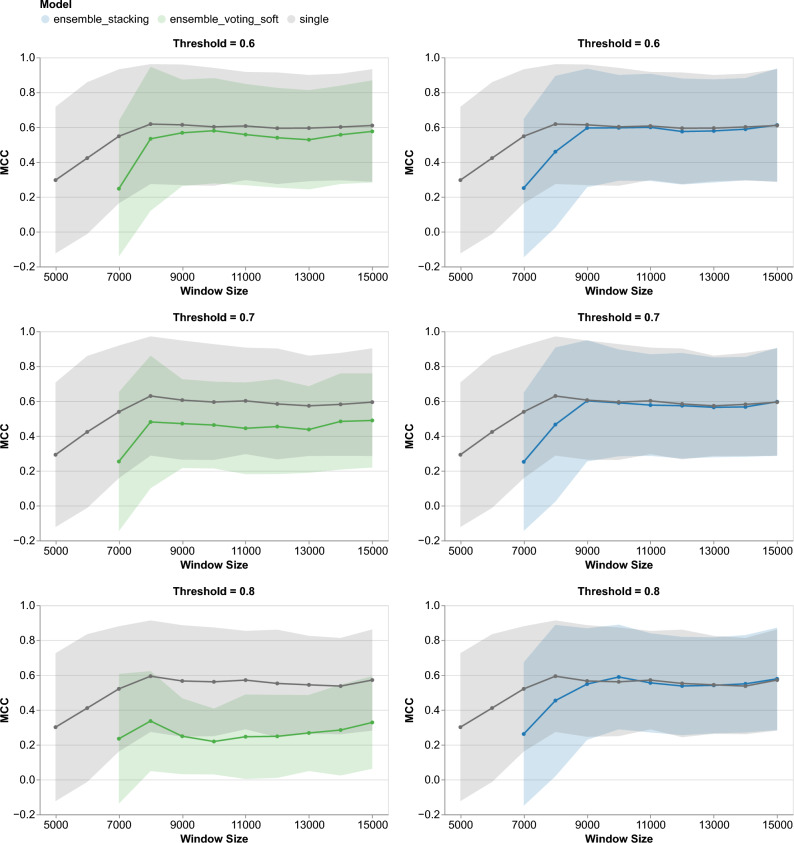
Comparison of Matthew correlation coefficient (MCC) between the single and ensemble classifiers, both using default hyperparameters, evaluated on the genomic island (GI) boundaries prediction task with different thresholds and window sizes. The evaluation was performed using an existing pipeline developed by Banerjee et al. on 20 species in the Benbow test dataset^24^. Solid lines show the mean of MCC of GI boundaries prediction, whereas the shaded bands represent the standard deviation across 20 species.

Figure 7 shows the comparison of MCC between the single and the ensemble classifiers, both using default hyperparameters. At a threshold of 0.8, the ensemble classifier with *voting_soft* performed much worse than the single classifier. While lowering the threshold led to a marginal improvement in the ensemble classifier’s performance, it failed to bridge the performance gap. This indicates that the ensemble classifier with *voting_soft* is sensitive to threshold calibration and its performance is fundamentally inferior to the single classifier. In contrast, ensemble *stacking* demonstrated performance parity with the single classifier at threshold 0.8. This similarity remained consistent as the threshold was reduced.

While changing the threshold affected the ensemble classifiers differently, we noticed that both ensemble classifiers failed to predict boundaries with window sizes of 5,000 bp and 6,000 bp. Both ensemble classifiers were just able to capture the signal with a 7,000 bp window. Their performance increased as the window sizes increased and became steady starting from either window sizes of 9,000 bp or 10,000 bp, depending on the threshold. As an exception, ensemble *voting_soft* performance remained between 0.2 and 0.33 in MCC. Overall, ensemble *voting_soft* could not reach the performance of the single classifier in all selected windows, whereas ensemble *stacking* performance could reach the single classifier starting from a window size of 9,000 bp.

**Fig. 8:**
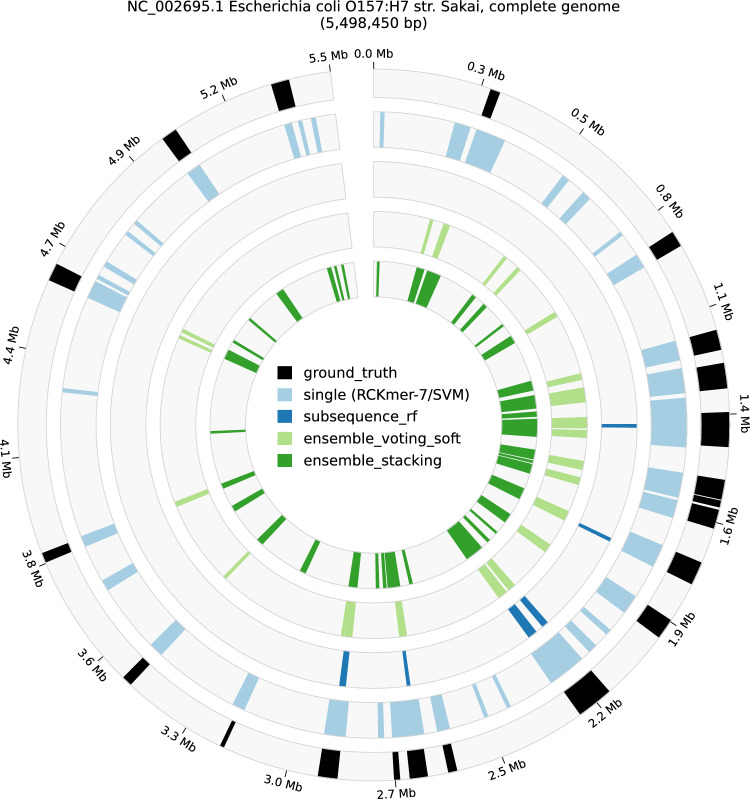
A circular plot depicting the genomic islands (GIs) on *Escherichia coli* O157:H7 str. Sakai (NC_002695.1). The plot shows GIs predicted by different classifiers and also the ground truth derived from previous literature study collected by Langille et al.^25^.

In addition to the main evaluation, we compared predicted GIs boundaries from all classifiers compared with the ground truth on *Escherichia coli* O157:H7 str. Sakai (NC_002695.1), depicted in Fig. 8. Compared with the ground truth, the best single classifier suggested more GIs boundaries than the ones in the ground truth. Subsequence/random forest was only able to capture a few GIs without providing potential GIs. The ensemble *voting_soft* reduced the predictions from the single classifier and thereby missed the positives from the ground truth. The predictions from ensemble *voting_soft* were mostly a subset of the best single classifier, whereas the ensemble *stacking* could suggest a novel GIs, for instance, the region between 4,1 Mb and 4,4 Mb that is slightly different from the prediction from the best single classifier. Interestingly, the predicted GIs from the best single classifier occupied larger regions for the same GIs in the ground truth, suggesting there might be some signal supporting the integration of GIs into the genome. All predictions were obtained using the pipeline in^24^ with window size of 10,000 bp and threshold 0.8.

## Discussion

In this study, we examined the efficacy of combining different data representations in ensemble learning for the detection of horizontal gene transfer (HGT), modeled as a classification task. We used a two-stage ensemble selection strategy and various ensemble methods and solutions to create ensemble classifiers. Aligned with previous studies^15,19,26^, our study demonstrated that an ensemble classifier that integrated low-correlated data representations RCKmer-7 and Subsequence) outperformed individual representations (RCKmer-7), better in capturing more (see Supplementary Fig. S3). This finding validated our hypothesis that low-correlated data representations can capture complementary GI information^9^. Nevertheless, further analysis is required to elucidate the precise information captured by these data representations. This analysis could open the way to leveraging explainable artificial intelligence (XAI), which is beyond the scope of this study. Furthermore, our analysis revealed a statistical difference in the performance of heterogeneous and homogeneous base classifiers in ensemble classifiers, with the exception of random forest and SVM. This outcome is attributed to the inherent characteristics of random forest, as it is already an ensemble classifier, whereas SVM alone has been demonstrated to exhibit superior performance.

We examined the performance of the ensemble classifier in localizing GI boundaries using different thresholds and window sizes. We identified a discrepancy between the evaluation results of the classification and boundary prediction tasks. Despite its superior performance in the classification task, the representative ensemble classifier (RCKmer-7/SVM and Subsequence/RandomForest with the *voting_soft* strategy) failed to perform in the boundaries prediction task. As an alternative, we changed the ensemble method to *stacking*.

Ensemble with *voting_soft* is sensitive to the threshold selection compared with the *stacking* ensemble and single classifiers. We then hypothesized that this sensitivity occurred because *voting_soft* uses raw probability from each candidate. Aggregating these probabilities would reduce the final probability and thus, it could not pass a certain threshold. This was evidenced by the increase in performance when the threshold was lowered. In contrast, the *stacking* ensemble is more robust because it has a meta-learner that learns the best way to combine raw probabilities of candidates for the ensemble. Overall, we found that the ensemble classifiers are worse or at least as good as the single classifier. This suggests that although ensemble classifiers were shown to achieve higher Recall in the classification task, it could not be translated in GI boundaries prediction task. Moreover, this finding aligns with a previous study showing that a model trained on a classification task is not suited for a whole-genome scan task^20^.

In addition to the evaluation on the Benbow test dataset, we conducted the evaluation on another dataset consisting of GIs from 6 species reported in the literature^24^. The evaluation results corroborated those of the Benbow test dataset, demonstrating a discrepancy between classification and GI boundaries prediction tasks (see Supplementary Figs. S4, S5, and S6, and Supplementary Table S1). We assumed there could be an optimal solution for the pipeline settings and wanted to run cross-validation directly on the boundaries prediction task, but found it challenging because the pipeline involves not only classification but also other processes, such as sliding windows and refinement. Furthermore, the evaluation procedure for the boundaries prediction task differs from that of the classification task. The boundaries prediction task evaluates the number of overlapping predictions and the ground truth at the nucleotide level. In contrast, the classification task evaluates the number of correctly and incorrectly identified genomic segments. The failure of the ensemble classifier to translate the performance in GI boundaries prediction, despite its success in the classification task, underscores the limitation of the problem definition for ML approaches of GIs detection. Current ML approaches first train models on a classification task, then apply the models to predict GI boundaries within a genome. In the classification task, models are mainly trained to learn the characteristics of GIs by means of data representations, whereas the boundaries prediction task aims at finding the precise location of GIs within a genome. Using a sliding window approach might only capture some parts of GIs that may be mistakenly misclassified by the trained model. Moreover, the current evaluation procedure that calculates the exact overlapping of the predicted GIs and the ground truth at the nucleotide level might obscure the evaluation results. Furthermore, the absence of a reliable benchmark dataset with the precise location of GIs impedes the proper evaluation. Future studies may benefit from reformulating the GIs detection as a genome-wide scanning problem, or shift to a more holistic approach to understand how these islands were integrated into the genomes. Model development should go into a context-aware learning that can learn the context surrounding the GIs and not only learn the characteristics of GIs. This transition would necessitate collaborative efforts to develop proper benchmark datasets and evaluation frameworks, which currently exceed the scope of our study.

Although our study yielded very insightful findings, there are some limitations. First, we did not optimize the hyperparameters during the ensemble selection process since we focused on combining data representations. This could result in a different set of candidates for the ensemble classifier. Second, we applied only standard ensemble methods, including *voting_soft*, *voting_hard*, and *stacking*, as we focused on examining the effectiveness of combining multiple data representations. Other ensemble methods, including Bagging and HBoost^27^, could be considered in the future to maximize the ensemble performance. Third, we did not explore other models besides Logistic Regression for the meta- learner in ensemble learning with *stacking*. The performance of ensemble classifiers is affected by different combinations of meta learners and base learners, but using a more complex model would lead to overfitting, especially with a small dataset^28^; thus, further research could explore the impact of different meta learners. Fourth, while we demonstrated that the ensemble classifier outperformed the single classifier in the classification, the finding is limited to the dataset. We did not conduct our study on multiple datasets because finding another reliable datasets for GIs detection is challenging. This highlights a pressing need for the scientific community to craft a gold standard dataset to further validate our findings. Fifth, while we observed overall improvement on the ensemble classifier, the improvement varied on different species, with some of them experiencing a drop in MCC (see Fig. 6b). These variations could arise from class imbalances in training or test datasets, and the biological diversity present across multiple bacterial lineages. However, this study does not extensively examine species-level differences. Therefore, this study advocates for the importance of curating taxonomically diverse, multi-species datasets in future research to differentiate model learning behavior from inherent biological signals.

## Conclusion

Our study aims to examine the value of combining different data representations in ensemble learning for HGT detection. Combining different data representations, especially those with low correlation, in an ensemble learning results in a superior performance. Our results highlight that an ensemble classifier can capture more positives compared to a single classifier; however, the difference is not statistically significant. Aligned with a previous study^20^, we validated that a model trained on a classification task is not suitable for a whole-genome scan task, evidenced by the performance discrepancy between the classification task and the boundaries prediction task. This finding highlights the need to revisit the problem formulation for HGT detection, for instance, as a whole-genome scan task or a regression task, and motivates the development of more advanced approaches. Our study shows that the current pipeline for HGT detection remains useful, but it requires careful application to avoid misinterpretation.

## Methods

### Problem definition

The detection of GIs involves two main tasks: genomic segment classification and boundaries prediction. The classification tasks classify genomic segments into GIs (positives) or non-GIs (negatives), while boundaries prediction determines the specific locations of these GIs within a genome. We trained diverse ML classifiers for the classification task. Then, we applied the trained classifiers to predict GI boundaries in a genome. Although GIs encompass a variety of MGEs, this study narrows its scope to GIs defined as distinct chromosomal segments obtained via HGT. This approach excludes non-integrative MGEs, such as plasmids.

### Datasets

In this study, we used a dataset, called Benbow dataset, from a previous study^24^. This dataset comprises sequences of GIs (positives) and non-GIs (negatives) from 167 bacterial genomes, 147 genomes for training, and the remaining 20 genomes for testing. In total, the dataset has 3135 sequences. 1742 sequences belong to the positive class and 1393 to the negative class with a mean length of 15861±22048 bp and 14256±8404 bp, respectively. Benbow training dataset consists of 2569 sequences (1329 positives and 1240 negatives) and Benbow test dataset includes 566 sequences (413 positives and 153 negatives) We used a Python package, iLearnPlus v1.5^29^, to transform the sequences into various data representations. In total, we selected 44 data representations (see Supplementary Table S2). The complete genomes of the species in the test dataset were used for evaluating the model’s effectiveness in localizing GIs within the full genomes. Additionally, we used a literature dataset^24^, containing curated GIs from 6 species reported in literature. Table 5 shows the 6 species in literature dataset 4.

**Table 4:** List of species in Benbow test dataset.

Accession number	Organism	#samples (+/-)	Proportion (%)
NC_007606.1	*Shigella dysenteriae* Sd197	19 (14/5)	3.35
NC_007958.1	*Rhodopseudomonas palustris* BisB5	22 (21/1)	3.88
NC_008321.1	*Shewanella sp.* MR-4	13 (11/2)	2.29
NC_008563.1	*Escherichia coli* APEC O1	23 (17/6)	4.06
NC_009665.1	*Shewanella baltica* OS185	26 (20/6)	4.59
NC_009708.1	*Yersinia pseudotuberculosis* IP 31758	16 (15/1)	2.82
NC_009783.1	*Vibrio campbellii* ATCC BAA-1116 chromosome I	31 (21/10)	5.47
NC_010501.1	*Pseudomonas putida* W619	63 (53/10)	11.13
NC_009512.1	*Pseudomonas putida* F1	67 (52/15)	11.83
NC_009438.1	*Shewanella putrefaciens* CN-32	23 (19/4)	4.06
NC_010322.1	*Pseudomonas putida* GB-1	67 (53/14)	11.83
NC_008253.1	*Escherichia coli* 536	23 (12/11)	4.06
NC_004603.1	*Vibrio parahaemolyticus* RIMD 2210633 chromosome 1	29 (21/8)	5.12
NC_010380.1	*Streptococcus pneumoniae* Hungary19A-6	11 (7/4)	1.94
NC_010515.1	*Burkholderia orbicola* MC0-3 chromosome 2	68 (56/12)	12.01
NC_010334.1	*Shewanella halifaxensis* HAW-EB4	22 (9/13)	3.88
NC_010473.1	*Escherichia coli* str. K-12 substr. DH10B	13 (2/11)	2.29
NC_004070.1	*Streptococcus pyogenes* MGAS315	4 (2/2)	0.7
NC_007432.1	*Streptococcus agalactiae* A909	17 (7/10)	3
NC_009504.1	*Brucella ovis* ATCC 25840 chromosome II	9 (1/8)	1.59

**Table 5:** List of species in literature dataset.

Accession number	Organism	#samples (+/-)	Proportion (%)
NC_002695.1	*Escherichia coli* O157:H7 str. Sakai	66 (24/42)	25.19
NC_003198.1	*Salmonella enterica* subsp. enterica serovar Typhi str. CT18	63 (18/45)	24.04
NC_003923.1	*Staphylococcus aureus* subsp. aureus MW2	30 (7/23)	11.45
NC_004070.1	*Streptococcus pyogenes* MGAS315	21 (6/15)	8.01
NC_004431.1	*Escherichia coli* CFT073	58 (13/45)	22.14
NC_004603.1	*Vibrio parahaemolyticus * RIMD 2210633 chromosome 1	24 (12/12)	9.16

### Experimental workflow

**Fig. 9:**
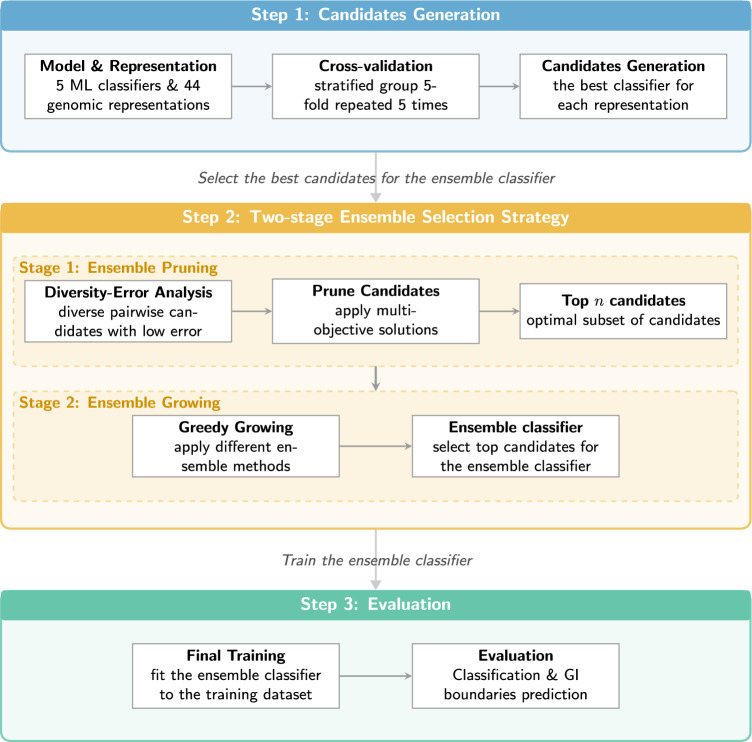
Flowchart of the experimental workflow for selecting and evaluating ensemble classifiers.

Our study employed a three-step framework, as illustrated in Fig. 9. First, we generated candidates for the ensemble classifier. Second, the generated candidates were selected by employing a two-stage ensemble selection strategy. Third, we trained an ensemble classifier with the selected candidates and evaluated it on classification and GI boundaries prediction tasks.

### Step 1: Candidates generation

#### Model and representation

We trained 5 base classifiers—Naïve Bayes, logistic regression (LR), decision tree (DT), support vector machine (SVM), and random forest (RF)—from scikit-learn v1.7.2^30^ using default hyperparameters to focus on evaluating the impact of data representations rather than optimizing model-specific parameters. This approach was intended to minimize complexity and maintain consistency across different models. Each base classifier is trained with 44 data representations, resulting in 220 combinations of representation and classifier.

#### Cross-validation

Similar to our previous study^9^, we employed stratified group 5-fold cross-validation for each combination of data representations and classifiers to ensure reliable performance, but we repeated the cross-validation 5 times. A pre-defined cross-validation split is computed to ensure that the cross-validation split is the same for all combinations. Each fold is composed of 80% of the data for training and 20% for testing. The training data is further split into 80% of the data for training and 20% for validation. At the end, each fold consists of 64% of the data for training, 16% for validation, and 20% for testing. Additionally, we stored the predictions on the validation and testing dataset of each fold for all combinations of representations and classifiers. These predictions were then used in the two-stage ensemble selection strategy.

#### Candidates generation

To cope with the computational complexity, we limited each representation to one classifier that performs the best with the corresponding representation. Additionally, this prevents combining the same representation with different classifiers. At the end, we have 44 combinations of representation and classifier, which we refer to as candidates for the ensemble classifiers.

### Step 2: Two-stage ensemble selection strategy

#### Diversity-Error analysis

Diversity is one of the key properties of ensemble learning since the power of ensemble learning heavily depends on the diversity of the base classifiers^31^. Several metrics have been proposed to measure the diversity of an ensemble learning model, such as Q-statistics^32^ and Cohen’s kappa agreement^21^. In this study, we did not compare the effect of different diversity metrics as we focused more on understanding whether ensemble learning can improve the detection of HGT. Therefore, we chose only one diversity metric, namely Cohen’s kappa agreement, to measure the diversity between candidates.

Cohen’s kappa agreement ($\kappa$) measures the agreement between two classifiers beyond chance. For two classifiers $C_1$ and $C_2$, $\kappa$ is defined as $\kappa = \frac{p_o - p_e}{1 - p_e}$. $p_o$ is the *observed agreement*, *i.e.*, the proportion of instances on which both classifiers agree. $p_e$ is the *expected agreement by chance*, calculated from the marginal probabilities of the classifiers’ predictions.

Formally: $p\_o = \frac{N_{11} + N_{00}}{N}$ and $p\_e = \frac{(N_{11} + N_{10})(N_{11} + N_{01}) + (N_{01} + N_{00})(N_{10} + N_{00})}{N^2}$, where:

- $N_{11}$ = number of instances both classifiers predicted as positive,
- $N_{00}$ = number of instances both predicted as negative,
- $N_{10}$ = number predicted positive by $C_1$ but negative by $C_2$,
- $N_{01}$ = number predicted negative by $C_1$ but positive by $C_2$,
- *N* = total number of instances.

A $\kappa$ value of 1 indicates perfect agreement, 0 indicates agreement equivalent to chance, and negative values indicate less-than-chance agreement. In the diversity-error analysis, we used the predictions from the cross-validation in Step 1 for all folds. For each fold, we made all possible pairs of candidates. Then, we measured the $\kappa$ value for each pair based on the predictions of each candidate in the corresponding pair and calculated the average error rate of each candidate. We defined the error rate using Matthew correlation coefficient (MCC) as $1-MCC$.

#### Stage 1: Ensemble pruning

Ensemble pruning aims to select the optimal subset of candidates for the ensemble classifier, and it is formulated as a multi-objective optimization problem, with the objectives of maximizing candidates diversity (minimizing $\kappa$) and minimizing the candidates error rate.

To analyze the trade-off between diversity and performance, we constructed a kappa–error diagram^33^, where each point represents a pair of candidates illustrated in Fig.  2. The x-axis corresponds to $\kappa$ (agreement), and the y-axis corresponds to the average classification error rate of the two candidates. Classifier pairs that exhibit both low $\kappa$ (high diversity) and low error rate are located in the lower-left region of the diagram, indicating they are both accurate and diverse.

To select the optimal subset of candidates pairs, we applied two pruning solutions: Pareto frontier and convex hull as in Spänig et al.^15^. The pfront identified candidates pairs for which no other pair achieved both lower kappa and lower error rate, representing non-dominated trade-offs between diversity and performance. The chull retained pairs lying on the convex boundary of the kappa–error diagram, representing extreme optimal solutions. The optimal subset of candidates pairs is likely to include redundant classifiers. We filtered the selected candidates for the ensemble classifier by performing the following steps :

1. Count the number of times each candidates pair is selected across folds
2. Select top *m* ($m=40$) candidates pairs
3. Count the number of times each candidate is selected in the top (*m*) candidates pairs
4. Select top *n* ($n=20$) candidates

This ensures that the pruned candidates pairs are robust across cross-validation folds, and so are the unique candidates. In the ensemble pruning, we limit the maximum number of candidates permitted in the ensemble to 20, but not all candidates are included in the final ensemble classifier. In addition to Pareto frontier and convex hull, Spänig et al.^15^ selected best base classifiers as candidates for the ensemble classifier.

#### Stage 2: Ensemble growing

From the optimal subsets of candidates for the ensemble classifier identified in Stage 1, we employed an ensemble growing procedure to select the best-performing combination of candidates across cross-validation folds:

1. Start with an empty ensemble.
2. Iteratively add a candidate from the pruned subsets.
3. At each step, select the candidate whose inclusion yields the largest improvement in model performance.
4. Stop when no further improvement is observed or a predefined size limit is reached.

This greedy growing approach ensures that the final ensemble is both compact and high-performing, leveraging the most complementary classifiers identified during pruning.

During this process, we did not retrain each set of candidates, but we used each candidate’s predictions on the testing dataset from each fold. Then, we tested three ensemble methods to merge these predictions, namely:

- *voting_hard* assigns the final class label predicted by the majority of base classifiers.
- *voting_soft* averages predicted class probabilities and selects the class with the highest mean probability.
- *stacking* uses a meta learner to combine the predicted probabilities from each candidate; we trained a logistic regression with default hyperparameters from scikit-learn v1.7.2 as the meta learner on the validation dataset of each fold, then evaluated it on the testing dataset of the corresponding fold.

We ran the above steps for each cross-validation fold and reported the cross-validation results from three ensemble methods (*voting_hard*, *voting_soft*, and *stacking*) combined with three multi-objective solutions (pfront, chull, and best). Then, the optimal ensemble classifier was identified based on the cross-validation results by selecting one that outperforms the top-ranking single classifier.

##### Ensemble classifier selection

To select candidates for ensemble classifiers, we followed the selection procedure called selection by accuracy and diversity (SAD)^31^, and performed this procedure on each cross-validation fold as follows:

1. Calculate the performance of each candidate on the validation dataset.
2. Create unique pairs of candidates.
3. Compute the diversity of each pair of candidates.
4. Apply the two-stage ensemble selection strategy to select the optimal subset of candidates for the ensemble classifier.

After obtaining the optimal candidates for the ensemble classifier, we then trained this ensemble classifier on the entire Benbow training dataset before evaluating it on the Benbow test dataset. Particularly, we used ensemble classifiers from scikit-learn v1.7.2 (*VotingClassifier* with soft and hard voting, and *StackingClassifier* using a logistic regression with default hyperparameters (penalty=’l2’, C=1.0, solver=’lbfgs’) as a meta learner).

### Step 3: evaluation

We evaluated our study on two tasks, namely classification task and boundaries prediction task. The classification task aims to measure the performance of a classifier in classifying genomic segments into GI or non-GI, positive or negative classes, respectively. The boundaries prediction task assesses the performance of a classifier in localizing GIs in a genome. In our study, we adopted an existing pipeline developed by Banerjee et al.^24^ and evaluated the performance following Bertelli et al. and Banerjee et al.^24,34^, where: (i) true positive (TP): the number of nucleotides in the positive prediction that overlaps with positive reference data. (ii) true negative (TN): the number of nucleotides outside the positive prediction that overlaps with negative reference data. (iii) false positive (FP): the number of nucleotides in the positive prediction that overlaps with negative reference data. (iv) false negative (FN): the number of nucleotides outside the positive prediction that overlaps with positive reference data. We reported different evaluation metrics: F1 score, Matthew correlation coefficient (MCC), precision, recall, and accuracy. However, we mainly used MCC as the metric to report the analysis on every stage of the study.

## Supplementary Information

Supplementary Information.

## Data Availability

All data and code used for this study are available at https://github.com/andrejw27/ensemble-data-representations-for-hgt-detection
