# Supplementary material for: Evaluating ensemble learning approaches for horizontal gene transfer detection
Source: Sci Rep. 2026 May 28;16:16582. doi: 10.1038/s41598-026-53037-x (PMC13219461; doi:10.1038/s41598-026-53037-x)
Supplement: Supplementary file 1 — Supplementary Information. [file 41598_2026_53037_MOESM1_ESM.pdf]

Evaluating Ensemble Learning Approaches for Horizontal Gene Transfer Detection

Andre Jatmiko Wijaya[1,2], Aleksandar Anžel[1], and Georges Hattab[1,2,*]

[1]Robert Koch Institute, Center for Artificial Intelligence in Public Health Research (ZKI-PH), Berlin, 13353, Germany
[2]Freie Universität, Department of Mathematics and Computer Science, Berlin, 14195, Germany
[*]HattabG@rki.de

Supplementary Figures

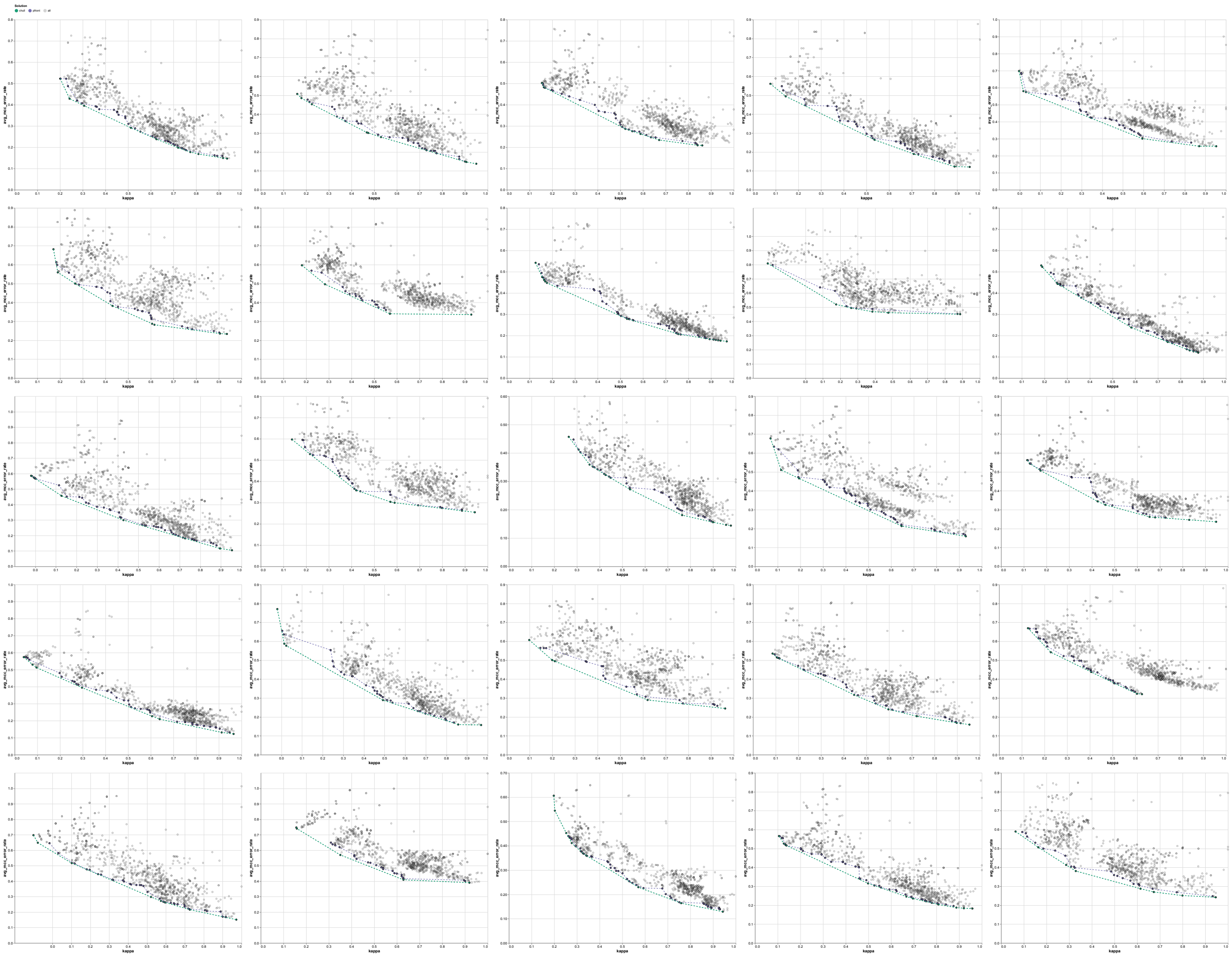

Figure S1. Kappa-error diagrams from all cross-validation folds. Each dot represents a pair of candidates (one data representation and one classifier). The gray dots correspond to all possible pairs of candidates. The green dots represent pairs selected by the convex hull, and the purple dots represent pairs selected by the Pareto frontier. The optimal solution for the ensemble classifier should have a low error rate and low Kappa, indicating high diversity, and is located in the lower left part of the diagram.

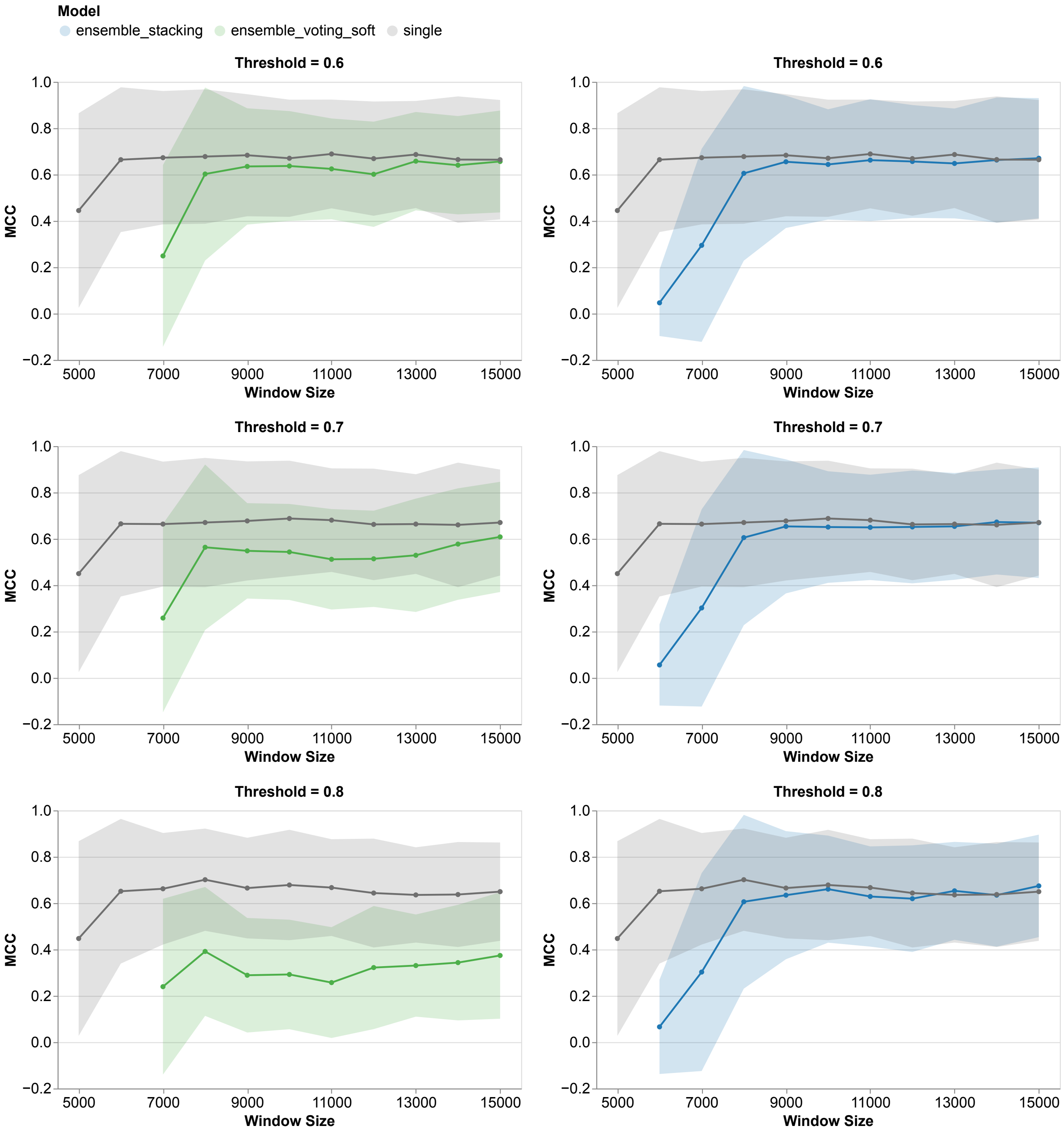

Figure S2. Comparison of Matthew Correlation Coefficient (MCC) between the single and ensemble classifiers with fine-tuned hyperparameters across 20 species in the Benbow test dataset[1], evaluated on the genomic island (GI) boundaries prediction task with different thresholds and window sizes. The single classifier was RCKmer-7/SVM and the ensemble classifier used RCKmer-7/SVM and Subsequence/Random Forest. Both used fine-tuned hyperparameters on the RCKmer-7/SVM. Solid lines show the mean of MCC of GI boundaries prediction, whereas the shaded bands represent the standard deviation across 20 species.

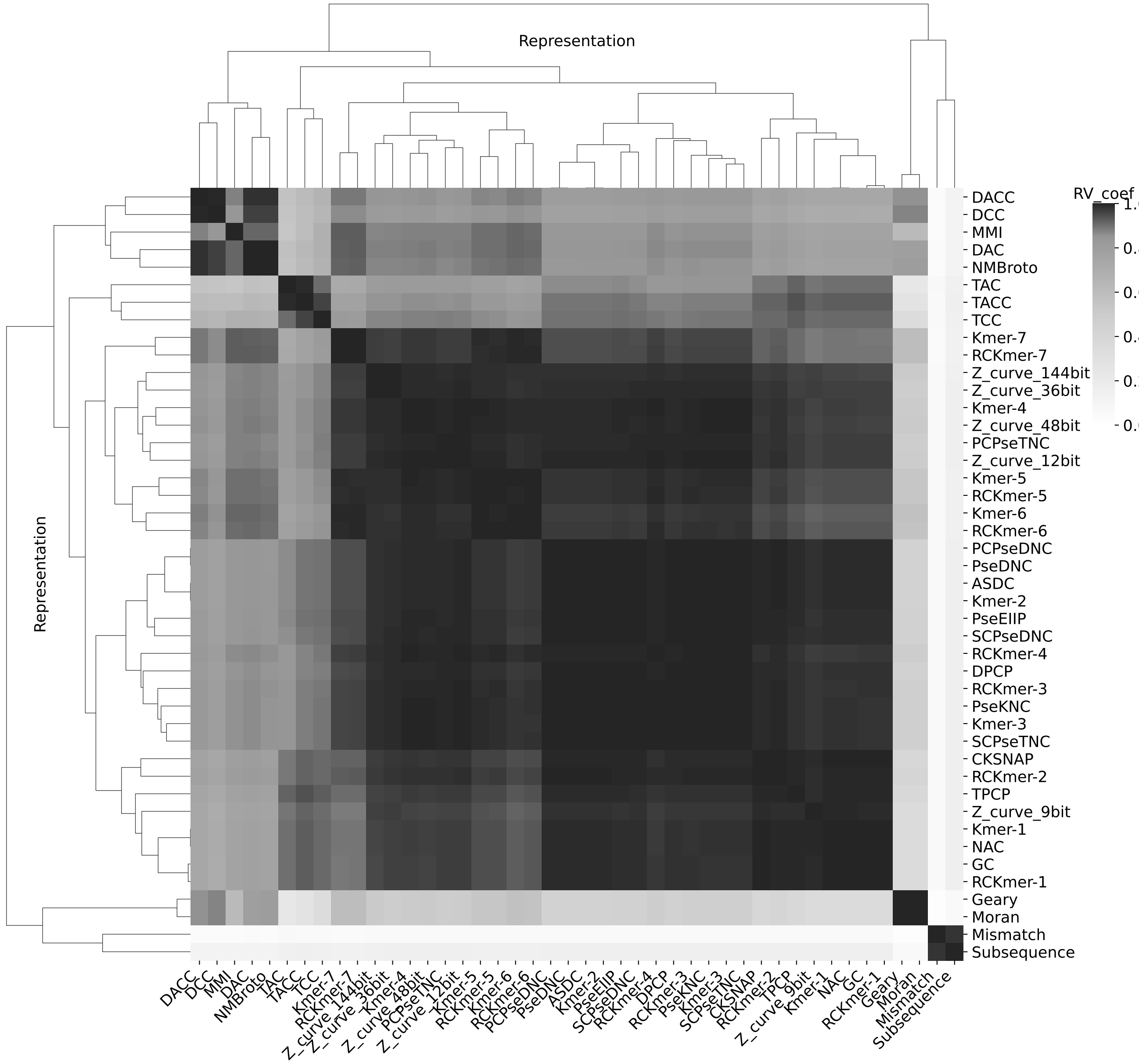

Figure S3. Correlation between data representations based on the adjusted RV-coefficient (example: Benbow data set). Color coding corresponds to the adjusted RV-coefficient, where a darker color represents a higher correlation between data representations. Adapted from "Genomic Data Representations for Horizontal Gene Transfer Detection" by Wijaya *et al.*, 2025, *NAR Genomics And Bioinformatics*. **7** (2025,11), https://doi.org/10.1093/nargab/lqaf165

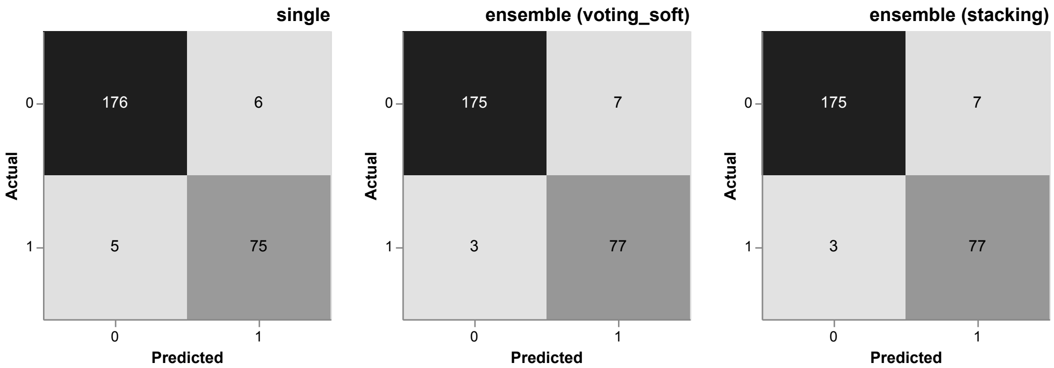

(a) Confusion matrices comparing the single classifier, ensemble classifier with *voting_soft*, and *stacking* with default hyperparameters

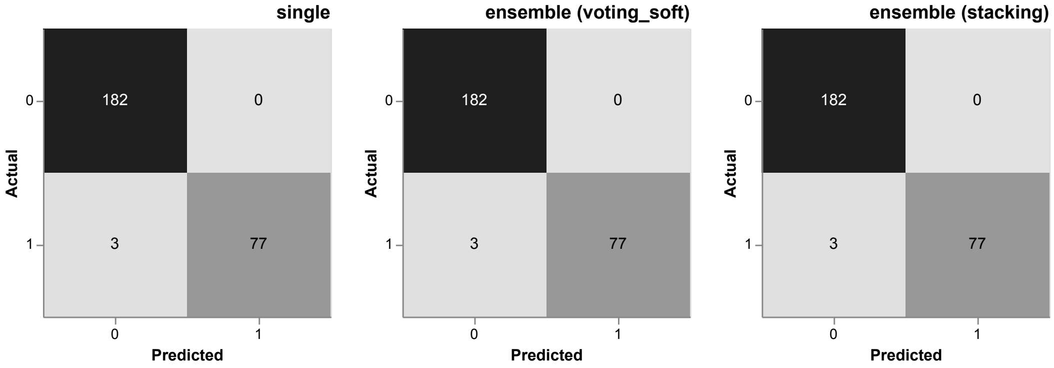

(b) Confusion matrices comparing the single classifier, ensemble classifier with *voting_soft*, and *stacking* with fine-tuned hyperparameters

Figure S4. Comparison of single classifiers with ensemble classifiers, specifically *voting_soft* and *stacking*, using a literature dataset[1]. This is illustrated through confusion matrices for cases with default and fine-tuned hyperparameters in (a) and (b), respectively. In this context, class 0 is defined as negative and class 1 as positive. The fine-tuned hyperparameters were applied solely to the RCKmer-7/SVM classifier in the single and ensemble classifiers, while Subsequence/Random Forest for the ensemble used default hyperparameters. When using default hyperparameters, ensemble classifiers identified 2 additional positive cases but missed 1 negative case. Conversely, no discrepancies were noted among the classifiers when fine-tuned hyperparameters were utilized.

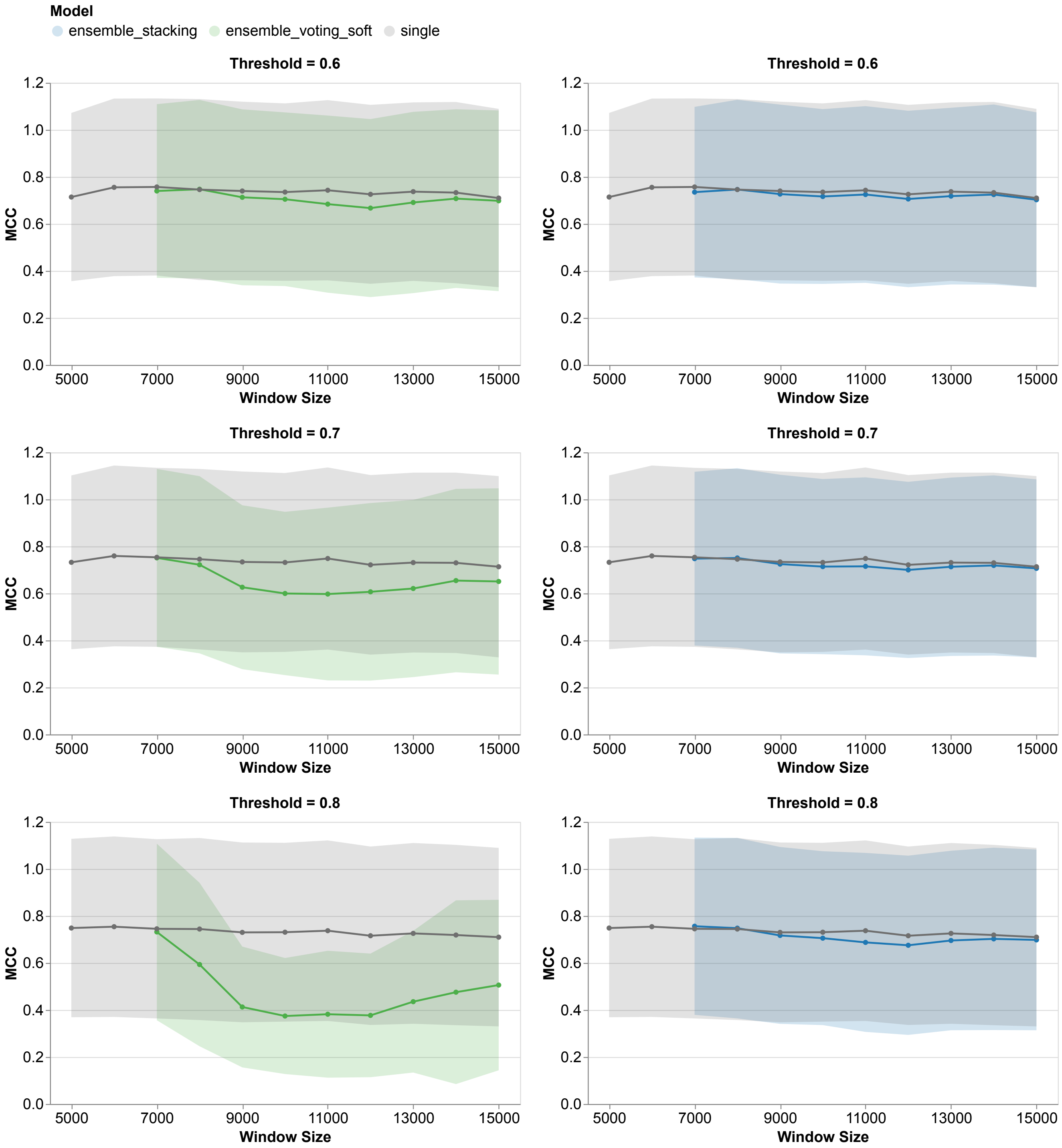

Figure S5. Comparison of Matthew Correlation Coefficient (MCC) between the single and ensemble classifiers with default hyperparameters across 6 species in the literature dataset[1], evaluated on the genomic island (GI) boundaries prediction task with different thresholds and window sizes. The single classifier was RCKmer-7/SVM and the ensemble classifier used RCKmer-7/SVM and Subsequence/Random Forest. Solid lines show the mean of MCC of GI boundaries prediction, whereas the shaded bands represent the standard deviation across 6 species.

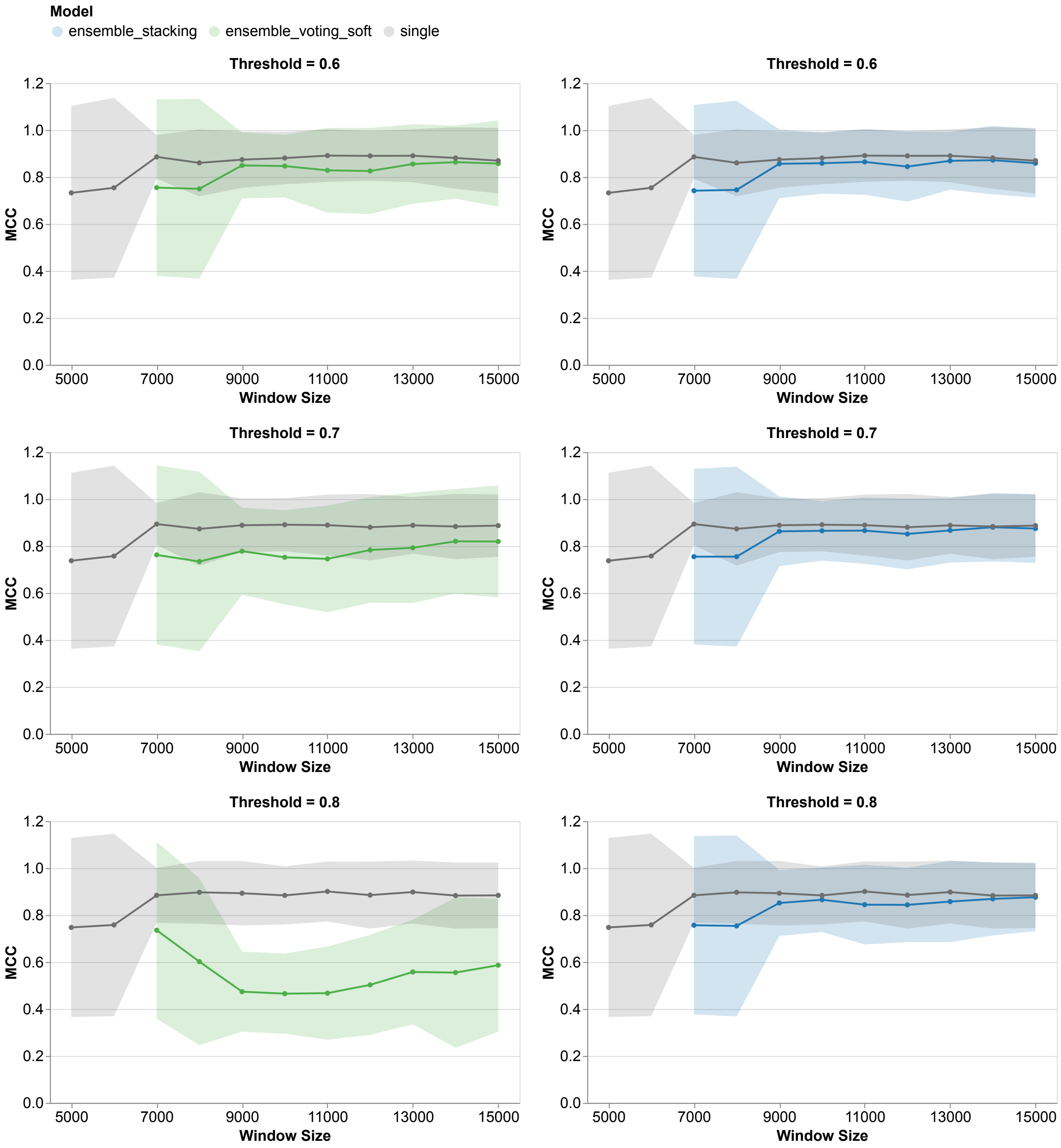

Figure S6. Comparison of Matthew Correlation Coefficient (MCC) between the single and ensemble classifiers with fine-tuned hyperparameters across 6 species in the literature dataset[1], evaluated on the genomic island (GI) boundaries prediction task with different thresholds and window sizes. The single classifier was RCKmer-7/SVM and the ensemble classifier used RCKmer-7/SVM and Subsequence/Random Forest. Both used fine-tuned hyperparameters on the RCKmer-7/SVM. Solid lines show the mean of MCC of GI boundaries prediction, whereas the shaded bands represent the standard deviation across 6 species.

Supplementary Tables

Table S1. Evaluation metrics achieved by the single and ensemble classifiers (with *voting_soft* and *stacking*) as well as other baselines for GIs detection are listed by descending Matthew Correlation Coefficient (MCC). Each metric is averaged across 6 species in the literature dataset[1]. All evaluation results of other baselines are available in `https://github.com/FriedbergLab/GenomicIslandPrediction/tree/master/evaluation/predictions`[1]

Predictor	MCC	F1 score	Precision	Recall	Accuracy
Single (fine-tuned)*	**0.88**	**0.94**	0.98	**0.92**	**0.94**
Ensemble (*stacking* fine-tuned)*	0.86	0.93	0.98	0.90	0.92
Single	0.73	0.78	0.99	0.74	0.85
Ensemble (*stacking*)	0.70	0.77	0.99	0.72	0.84
IslandViewer4	0.68	0.79	0.99	0.67	0.82
TreasureIsland	0.58	0.73	0.96	0.59	0.76
Islandpath DIMOB	0.53	0.64	0.99	0.48	0.70
Ensemble (*voting_soft* fine-tuned)*	0.46	0.54	0.99	0.40	0.65
AlienHunter	0.40	0.64	0.75	0.57	0.70
Ensemble (*voting_soft*)	0.37	0.43	**1.00**	0.32	0.61
Islander	0.32	0.35	**1.00**	0.23	0.56
SIGI-HMM	0.27	0.31	**1.00**	0.20	0.55
IslandPick	0.23	0.26	**1.00**	0.16	0.52

*fine-tuned hyperparameters were only for RCKmer-7/SVM (kernel:'rbf', C: 2, gamma:'scale'), obtained by our previous study[?], but default hyperparameters for Subsequence/RF (n_estimators:100, criterion:'gini', max_samples_split:2, min_samples_leaf:1, max_features: 'sqrt').

Table S2. List of data representations.

Feature Group	Data Representation	Dimensionality
Nucleic acid composition	NAC[2]	4
	CKSNAP[2]	64
	Subsequence[3]	64
	Mismatch[3]	64
	ASDC[4]	16
	Z_curve_9bit[5]	9
	Z_curve_12bit[5]	12
	Z_curve_36bit[5]	36
	Z_curve_48bit[5]	48
	Z_curve_144bit[5]	144
	Kmer-1	4
	Kmer-2	16
	Kmer-3	64
	Kmer-4	256
	Kmer-5	1024
	Kmer-6	4096
	Kmer-7	16384
	RCKmer-1	2
	RCKmer-2	10
	RCKmer-3	32
	RCKmer-4	136
	RCKmer-5	512
	RCKmer-6	2080
	RCKmer-7	8192
	GC	1
Electron-ion interaction pseudopotentials	PseEIIP[6]	64
Autocorrelation and cross-covariance	DAC[7]	12
	DCC[7]	60
	DACC[7]	72
	TAC[7]	4
	TCC[7]	4
	TACC[7]	8
	Moran[8]	12
	Geary[9]	12
	NMBroto[10]	12
Physicochemical property	DPCP[11]	96
	TPCP[11]	768
Mutual information	MMI[12]	30
Pseudo nucleic acid composition	PseDNC[13]	19
	PseKNC[13]	67
	PCPseDNC[13]	19
	PCPseTNC[13]	67
	SCPseDNC[13]	34
	SCPseTNC[13]	70

References

1. Banerjee, P., Eulenstein, O. & Friedberg, I. Discovering genomic islands in unannotated bacterial genomes using sequence embedding. *Bioinformatics Advances*. **4** (2024), http://doi.org/10.1093/bioadv/vbae089
2. Chen, Z., Zhao, P., Li, F., Marquez-Lago, T., Leier, A., Revote, J., Zhu, Y., Powell, D., Akutsu, T., Webb, G. & Others iLearn: an integrated platform and meta-learner for feature engineering, machine-learning analysis and modeling of DNA, RNA and protein sequence data. *Briefings In Bioinformatics*. **21**, 1047-1057 (2020)
3. Liu, B., Gao, X. & Zhang, H. BioSeq-Analysis2. 0: an updated platform for analyzing DNA, RNA and protein sequences at sequence level and residue level based on machine learning approaches. *Nucleic Acids Research*. **47**, e127-e127 (2019)
4. Wei, L., Zhou, C., Chen, H., Song, J. & Su, R. ACPred-FL: a sequence-based predictor using effective feature representation to improve the prediction of anti-cancer peptides. *Bioinformatics*. **34**, 4007-4016 (2018)
5. Gao, F. & Zhang, C. Comparison of various algorithms for recognizing short coding sequences of human genes. *Bioinformatics*. **20**, 673-681 (2004)
6. Nair, A. & Sreenadhan, S. A coding measure scheme employing electron-ion interaction pseudopotential (EIIP). *Bioinformation*. **1**, 197 (2006)
7. Liu, B., Liu, F., Fang, L., Wang, X. & Chou, K. repDNA: a Python package to generate various modes of feature vectors for DNA sequences by incorporating user-defined physicochemical properties and sequence-order effects. *Bioinformatics*. **31**, 1307-1309 (2015)
8. Lin, Z. & Pan, X. Accurate prediction of protein secondary structural content. *Journal Of Protein Chemistry*. **20**, 217-220 (2001)
9. Sokal, R. & Thomson, B. Population structure inferred by local spatial autocorrelation: an example from an Amerindian tribal population. *American Journal Of Physical Anthropology: The Official Publication Of The American Association Of Physical Anthropologists*. **129**, 121-131 (2006)
10. Horne, D. Prediction of protein helix content from an autocorrelation analysis of sequence hydrophobicities. *Biopolymers: Original Research On Biomolecules*. **27**, 451-477 (1988)
11. Manavalan, B., Basith, S., Shin, T., Lee, D., Wei, L. & Lee, G. 4mCpred-EL: an ensemble learning framework for identification of DNA N4-methylcytosine sites in the mouse genome. *Cells*. **8**, 1332 (2019)
12. Wei, L., Su, R., Luan, S., Liao, Z., Manavalan, B., Zou, Q. & Shi, X. Iterative feature representations improve N4-methylcytosine site prediction. *Bioinformatics*. **35**, 4930-4937 (2019)
13. Liu, B., Liu, F., Wang, X., Chen, J., Fang, L. & Chou, K. Pse-in-One: a web server for generating various modes of pseudo components of DNA, RNA, and protein sequences. *Nucleic Acids Research*. **43**, W65-W71 (2015)
